# Leveraging AI for cell biology discovery

**DOI:** 10.1042/BST20253023

**Published:** 2026-01-08

**Authors:** Adriana Simizo, Mauro de Morais, Matheus Vesco, Helder Nakaya

**Affiliations:** 1 Hospital Israelita Albert Einstein, São Paulo, SP, Brazil; 2 Department of Clinical and Toxicological Analyses, University of São Paulo, São Paulo, SP, Brazil; 3 Institut Pasteur de São Paulo, São Paulo, SP, Brazil

**Keywords:** artificial intelligence, cell biology, deep learning, single-cell analysis, drug discovery, microscopy, protein structure prediction, synthetic biology

## Abstract

Artificial intelligence (AI) has become a transformative tool in cell biology, driving discoveries through the analysis of complex biological data. This review explores the diverse applications of AI, including its impact on microscopy, imaging, drug discovery, and synthetic biology. AI methods have significantly advanced our ability to analyze cellular images at single-cell resolution, uncover complex patterns in biological data, and predict cellular responses to various stimuli. Deep learning approaches have improved cell segmentation and tracking, facilitated precise single-cell transcriptomics analysis, and enhanced our understanding of protein structures and interactions. The application of AI to high-throughput technologies has also enabled detailed modeling of cell behavior. Key challenges are addressed, such as data quality requirements, model interpretability, and the need to democratize AI tools for broader accessibility in biology. Finally, the review considers future directions, highlighting AI’s potential to advance basic research and therapeutic applications.

## Introduction

Cellular systems represent one of the most complex and dynamic entities in nature, comprising intricate networks of molecular interactions that operate across multiple spatial and temporal scales. At any given moment, a single cell orchestrates thousands of biochemical reactions, maintains precise protein levels, coordinates organelle functions, and responds to environmental cues through elaborate signaling cascades. The complexity emerges not only from the vast number of components – including proteins, nucleic acids, metabolites, and lipids – but also from their nonlinear interactions and the emergent properties that arise from these relationships.

Understanding cellular behavior presents formidable challenges due to the sheer volume of molecular players involved, the speed at which cellular processes occur, and the remarkable adaptability of cells to changing conditions. Traditional experimental approaches, while valuable, often struggle to capture the full scope of these dynamic interactions, particularly when attempting to observe multiple cellular processes simultaneously or track rapid molecular events in real time [1,2]. This inherent complexity has historically limited our ability to fully comprehend cellular systems and accurately predict their behavior under various conditions.

Artificial intelligence (AI) has emerged as a transformative force in cell biology research, advancing the analysis of complex biological data and driving innovation. This transformation is particularly evident in deep learning algorithms, which enable powerful analysis of cellular images at single-cell resolution, allowing researchers to extract detailed information about cell morphology and molecular processes [3,4]. Beyond imaging analysis, advanced AI approaches are rapidly transforming both data acquisition and analysis in biological research, with modern AI tools being particularly effective at uncovering mechanistic relationships across multiple layers of cellular biology [5–9]. These advanced modeling capabilities extend to digital twins of human cells, where AI can predict cellular responses to various stimuli, streamlining drug discovery and enabling the testing of billions of molecular combinations *in silico* [10,11]. Also, the emergence of contextual AI models has enhanced our ability to understand protein function across different cell types and tissues, generating hundreds of thousands of context-aware protein representations that reflect cellular and tissue organization [12]. In cancer research, AI models are solving key challenges such as data integration with high intraindividual variability, quantifying cellular plasticity, and identifying causal networks in oncogenesis [13–16].

**Figure 1:**
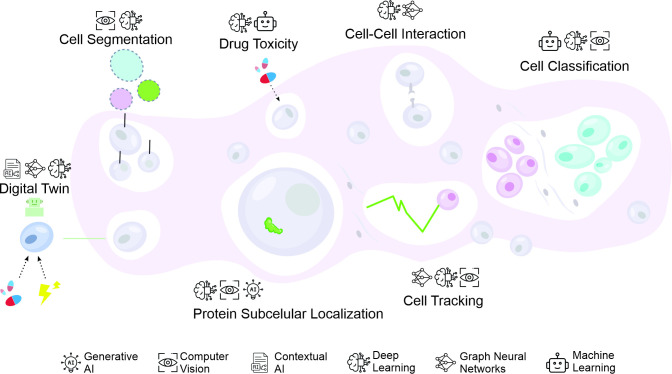
Overview of key artificial intelligence (AI) applications in cell biology. AI has significantly advanced seven major areas in cell biology: (1) cell segmentation, using deep learning for precise identification of individual cells; (2) drug toxicity prediction, through computational modeling of molecular and cellular responses; (3) cell–cell interaction analysis, employing network-based approaches to study cellular communication; (4) digital twin development, enabling *in silico* modeling of cellular systems; (5) protein subcellular localization prediction, using AI to determine protein distribution within cells; (6) cell tracking, for monitoring cellular dynamics over time; and (7) cell classification, providing automated identification of cellular phenotypes. The icons at the bottom represent the foundational AI methodologies driving these advancements.

This review focuses on the rapidly evolving landscape of AI applications in cell biology, ranging from microscopy to synthetic biology. The scope encompasses key areas of impact: imaging analysis, single-cell transcriptomics, protein structure prediction, and drug discovery. Figure 1 provides a visual summary of these key applications, highlighting how AI methodologies such as computer vision, graph neural networks, and generative AI are transforming research practices across these areas. In these domains, AI approaches can reveal biological interactions across molecular and cellular scales, from genomic regulation to protein function [17–19]. Recent advances include foundation models, improved imaging analysis tools, and novel computational platforms that enhance our understanding of cellular systems [20–23] (Table 1). These developments are essential for addressing the complexity of cellular systems, co-ordinating thousands of molecular interactions [43–45]. AI working alongside high-throughput technologies has advanced the modeling of cell behavior, driving progress in basic research and therapeutic applications [46]. While most reviews focus on specific AI applications, this work integrates advances across imaging, omics, protein structure prediction, and drug discovery, emphasizing key challenges and translational opportunities.

**Table 1:** Summary of artificial intelligence tools and their applications in cell biology.

**Tool**	**Function**	**Performance metrics**	**URL**
**Drug discovery and synthetic biology**			
AtomNet [24]	Prediction of molecule–protein binding	Hit rate 5–8%; validation ~90%; 318 targets with 16B compounds	https://www.atomwise.com/
DeepTox [25]	Pipeline for predicting chemical toxicity	AUC 0.86 (12 assays); Tox21 winner; 12.7k compounds	https://www.bioinf.jku.at/research/DeepTox/
MoleculeNet [26]	Molecular ML benchmark	>700k compounds; ROC-AUC >0.90 on Tox21/HIV	https://moleculenet.org/
**Hypothesis generation**			
FieldSHIFT [27]	Framework for generating biological hypotheses	1.4k domain pairs; GPT-4 success 58% vs 92% human; 74% validated gene overlap	https://huggingface.co/datasets/levinlab/neuroscience-to-dev-bio
**Microscopy and imaging**			
Cellpose [28]	Model for cell and nucleus segmentation	73% detection accuracy for cells; human-level segmentation with ~500 annotated cells	https://www.cellpose.org/
CellProfiler [29]	Feature extraction and segmentation	>12M cells processed; rand index = 0.919–0.976	https://cellprofiler.org/
Cytoself [30]	Model for protein localization features	Predicts subcellular localization for 88% of proteins; trained on 1.1M fluorescence images	https://github.com/royerlab/cytoself
DeepCell [31]	Nuclear and whole-cell segmentation	F1 = 0.82 (overall); trained on >1.3M cells	https://deepcell.org/
deepGPS [32]	Model for protein localization prediction	Accuracy 0.81; AUROC 0.83 (4-class); 62k images, 1.3k proteins	https://bits.fudan.edu.cn/opengps/home
StarDist [33]	Model for cell and nucleus segmentation	Average precision: 0.86 on real microscopy nuclei; robust in crowded images	https://stardist.net/
TrackMate [34]	Automated tracking	F1 > 0.96 for breast cancer cells and >0.99 for T cells; supports 2D/3D segmentation	https://imagej.net/plugins/trackmate/
U-Net [35]	Model for image segmentation	IoU up to 0.9 (near human-level accuracy) for 2D/3D cell segmentation	https://lmb.informatik.uni-freiburg.de/people/ronneber/u-net/
**Predicting cellular dynamics and behavior**			
NETISCE [36]	Cell fate reprogramming	85% accuracy across validated perturbations; remains >75% accurate under 50% simulated noise	https://github.com/VeraLiconaResearchGroup/Netisce
PERCEPTION [37]	Prediction of patient drug response	Trained on 488 cell lines; predictive for 33% FDA-approved drugs	https://github.com/ruppinlab/PERCEPTION
**Transcriptomics and omics integration**			
DeepTalk [38]	Model for cell–cell communication	AUC 0.84–0.95 for ligand–receptor pair prediction	https://github.com/JiangBioLab/DeepTalk
MIDAS [39]	Deep learning model for mosaic data integration	Outperforms 19 tools in multimodal integration; >185k cells	https://github.com/labomics/midas
MultiVI [40]	Model for multimodal data integration	Integrates RNA, ATAC, and protein data from >89k cells; Spearman *R* = 0.8–0.86 for imputation	https://scvi-tools.org/
scGNN [41]	Model for single-cell clustering	Outperforms 9 imputation and 4 clustering tools across 4 scRNA-seq benchmarks	https://github.com/juexinwang/scGNN
scGPT [6]	Generative AI for single-cell multi-omics	Trained and evaluated on 10.2M cells and 20–30% better temporal continuity than other existing tools	https://github.com/bowang-lab/scGPT
SpatialScope [42]	Spatial and single-cell transcriptomics integration	Integration across >1M spots; higher gene-level correlation (r ≈ 0.91)	https://github.com/YangLabHKUST/SpatialScope

## AI in microscopy and imaging

Deep learning approaches, including specialized architectures like U-Net [47] and Cellpose [28], have significantly improved cell segmentation and tracking. These AI models can effectively distinguish individual cells even in densely packed tissues and challenging imaging conditions, achieving accuracy rates above 90% [48]. Platforms like TrackMate [34] combine deep learning with segmentation algorithms, such as StarDist [33] and Cellpose [28], for robust tracking in transmitted light imaging. This combination has proven particularly valuable for long-term time-lapse imaging where phototoxicity must be minimized [49].

Beyond general cell and nucleus tracking, recent advances have enabled precise subcellular tracking and phenotyping. For example, deep learning-based frameworks have been applied to extract mitochondrial morphological and functional phenotypes from high-content images, providing insights into metabolic states and drug responses [50]. Similarly, AI approaches for spindle phenotype analysis allow detailed characterization of mitotic processes and structural variations at subcellular resolution [51]. These specialized trackers expand the scope of AI-powered imaging far beyond whole-cell analysis.

Automated phenotypic analysis, powered by machine learning models, classifies cell states and behaviors with high precision. Deep learning algorithms can identify complex morphological patterns and cellular states without requiring predefined features, allowing for unbiased detection of phenotypic variations [52–55]. These systems can process high-dimensional data from thousands of cells simultaneously, categorizing them based on morphology, behavior, and other characteristics [54–56]. Modern AI approaches are particularly effective at detecting subtle phenotypic changes that might be missed by traditional analysis methods, achieving up to 99% accuracy in phenotype prediction when combining both cellular and colonial morphological parameters [57].

Deep learning has transformed the mapping of protein subcellular localization through systems like cytoself, which can analyze the distribution of proteins across different cellular compartments without requiring preexisting annotations [30]. AI models with high precision in identifying organelles and protein locations have enabled detailed maps of cellular organization [7,58–62]. Furthermore, advanced computational approaches such as deepGPS [32] now achieve protein subcellular localization prediction directly from sequences, producing both textual annotations and artificial fluorescence images that quantitatively represent cellular and tissue organization. The use of these methodologies has facilitated the development of comprehensive protein localization atlases, systematically capturing diverse patterns of cellular organization [30].

Quantitative image analysis tools measure cellular features with precision, leveraging advanced automation and computational pipelines. These systems extract parameters such as size, shape, texture, and fluorescence intensity [35,54,63–65]. Modern AI approaches can analyze both bright-field and fluorescence images, providing detailed measurements of cellular structures without the need for invasive staining techniques [66,67]. Deep learning combined with high-throughput imaging allows the extraction of up to 17 distinct morphological features per cell, supporting comprehensive quantitative profiling of cellular populations [68]. These measurements can be performed in real time, enabling dynamic analysis of cellular changes during experiments [69,70]. The same approach can also be applied to detect and count human parasites, such as *Trypanosoma cruzi* from micrographs of blood smear samples, achieving 90.5% sensitivity and 88.6% specificity even when using low-resolution photos taken by smartphone cameras attached to microscopes [71].

## Transcriptomics and omics integration

Sophisticated deep learning approaches have transformed single-cell RNA sequencing (scRNA-seq) analysis, allowing precise identification of cell types and states. Advanced frameworks like scGNN [41] utilize graph neural networks to capture complex cell–cell relationships, achieving superior performance in gene imputation and cell clustering across diverse datasets. These models capture heterogeneous gene expression and predict cell trajectories by integrating regulatory signals [41]. Deep learning algorithms, such as scGPT, have improved the analysis of cellular dynamics, offering high performance in annotating cell types from scRNA-seq data [6,72].

The application of AI to spatial transcriptomics has transformed our understanding of tissue architecture and cellular interactions. SpatialScope [42], a unified deep generative model approach, enhances sequencing-based spatial transcriptomics data to achieve single-cell resolution while accurately inferring transcriptome-wide expression levels for image-based data. This technology facilitates the analysis of cellular communication and differentially expressed genes [42]. DeepTalk [38], an innovative method, uses graph attention networks to integrate scRNA-seq and spatial transcriptomics data, allowing accurate cell-type identification and insights into cell–cell communication.

**Figure 2:**
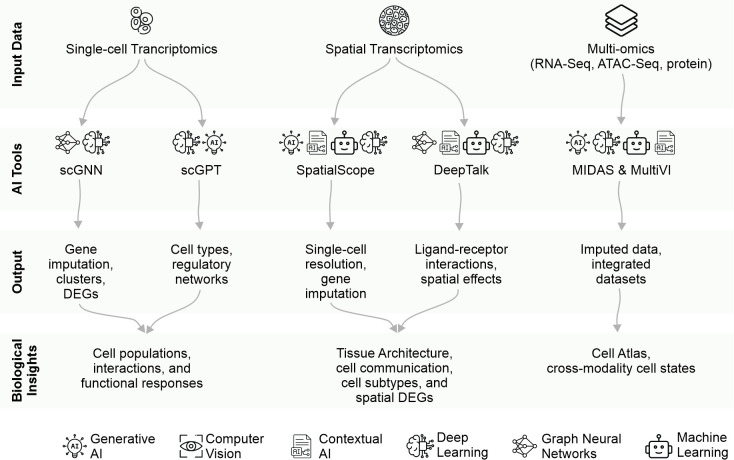
Artificial intelligence (AI) tools for transcriptomics and omics integration.

Advanced computational methods have emerged as powerful tools for comprehensive cellular analysis. MIDAS [39], a deep probabilistic framework, achieves simultaneous dimensionality reduction, imputation, and batch correction of mosaic data through self-supervised modality alignment. The framework enables flexible integration of different omics combinations and has demonstrated superior performance compared with 19 other methods [39]. Similarly, MultiVI from the scVI suite [40] provides a probabilistic model for analyzing multi-omic data, synthesizing information from different laboratories and technologies while maintaining biological relevance. These advanced AI approaches enable researchers to construct comprehensive cellular atlases and derive meaningful insights from complex biological systems [73]. A summary of these tools and functionalities is presented in Figure 2.

AI tools for single-cell transcriptomics, spatial transcriptomics, and multi-omics data are organized by their input data type, AI tool, output, and biological insights. For single-cell transcriptomics, scGNN enables gene imputation, clustering, and differential expression gene (DEG) analysis, while scGPT identifies cell types and regulatory networks. Spatial transcriptomics tools, such as SpatialScope and DeepTalk, provide single-cell resolution, ligand–receptor interaction analysis, and spatially resolved effects. Multi-omics integration is supported by tools like MIDAS and MultiVI, which generate imputed data and integrated datasets, enabling the construction of comprehensive cell atlases and cross-modality cell state analysis. The foundational methodologies driving these tools are represented by icons at the bottom.

## Predicting cellular dynamics and behavior

The cell cycle and signaling pathways represent intricate networks of molecular interactions operating across multiple scales. Cell signaling encompasses two essential processes: signal conduction for generation and intercellular transmission, and signal transduction for reception and processing of signals [74]. These pathways involve precise molecular events, including signal reception, amplification, distribution, and specific cellular responses that regulate cell behavior and function. Critical cellular determinations, such as cytoskeletal reorganization, cell cycle checkpoints, and programmed cell death, depend on stringent temporal regulation and specific spatial distribution of activated signal transducers [74].

The analysis of cellular signaling networks has significantly benefited from modern AI-based computational frameworks, which use non-parametric simulation techniques to provide accurate predictions without requiring detailed parameter knowledge [75]. Modern computational frameworks can simulate cell cycle progression through G1, S, G2, and M phases while incorporating checkpoint controls and regulatory mechanisms [76]. These systems employ various computational models, including cellular automata, Petri nets, Boolean models, rule-based systems, and artificial neural networks to analyze biological data [75]. During simulations, large volumes of data are generated, encompassing proteomics, genomics, interactomics, and metabolomics information, which serve as valuable input for predictive analytics [75].

Deep learning models can identify and predict cell fate decisions with remarkable accuracy. Using only bright-field images without artificial labeling, these models can identify differentiated cell types as early as 1 day of culture, achieving accuracy rates above 80% for various cell types [77]. AI systems have demonstrated success in predicting treatment responses in cancer, with models accurately forecasting how individual cells respond to both monotherapies and combination therapies [37,78]. The NETISCE computational tool [36] combines machine learning algorithms with signal flow analysis to estimate attractor landscapes and predict reprogramming targets, enabling the identification of cell fate transitions without requiring extensive experimental data.

AI-powered cellular dynamics modeling and fate prediction hold significant potential for advancing personalized medicine. These approaches enable precise matching of patients with effective drug treatments by analyzing scRNA-seq data [78]. AI models can also evaluate drug toxicity, identify specific molecular targets responsible for adverse effects, metabolic pathways, and potential clinical trial failures [79]. For cancer treatment, AI systems can analyze high-dimensional datasets to identify new dynamic diagnostic and prognostic biomarkers, leading to more accurate cancer care strategies [80]. These tools have proven particularly valuable in understanding tumor heterogeneity and predicting treatment responses across different cancer types [81].

## Applications in drug discovery and synthetic biology

Traditional drug discovery and toxicity assessment relied heavily on trial-and-error methods, where researchers tested compounds to identify therapeutic effects through extensive laboratory experiments. Animal studies served as the primary approach for toxicity evaluation, but these methods faced significant limitations due to cost, time, and ethical considerations. High-throughput screening techniques allowed researchers to screen large compound libraries, yet traditional methods often resulted in high failure rates, with 20% of drug candidates failing during clinical trials due to toxicity issues [82].

Various computational approaches have significantly improved drug toxicity prediction, marking a key advancement in pharmacological research. Machine learning algorithms can now evaluate drug toxicity using platforms like DeepTox [25] and MoleculeNet [26], which translate molecular structures into toxicity predictions. Advanced AI models combine chemical structure properties with protein target information to develop comprehensive toxicity scores, achieving high performance metrics (AUC = 0.83) and accuracy (ACC = 0.75) [82]. These systems can process thousands of compounds simultaneously, significantly reducing the time and cost of preclinical testing while maintaining high sensitivity (0.75) and specificity (0.74) [82].

Advancements in synthetic biology and computational tools have improved the design and optimization of cellular circuits for specific functions. These systems can predict the impact of bioengineering modifications on host cells and help create cells with enhanced properties such as increased productivity or stress resistance [83]. AI algorithms assist in dynamic pathway engineering by identifying architectures that optimize production and selecting optimal configurations for synthetic gene circuits [84,85]. These approaches enable the rapid construction and evaluation of engineered pathways, particularly in applications such as biofuel production and environmental remediation [84–86].

The pharmaceutical industry has witnessed significant growth in AI-driven companies. For example, Insilico Medicine has identified novel drug candidates, including a first-in-class fibrosis treatment using deep learning and generative adversarial networks [87]. Atomwise employs structure-based design via the AtomNet platform to predict small molecule binding, accelerating hit identification [24]. Exscientia has developed the first functional precision oncology platform using AI, with several AI-designed compounds currently in clinical development [88,89]. In personalized oncology, frameworks such as PERCEPTION have successfully predicted individual patient responses and resistance mechanisms to targeted therapies based on single-cell transcriptomic data [37]. AI is also being used to model protein–nanoparticle interactions. An example of this application is the prediction of the ‘protein corona’, which significantly affects nanoparticle behavior in biological environments. Accurate prediction of corona composition helps to optimize targeted delivery, minimize off-target effects, and enhance therapeutic efficacy [90].

## Hypothesis generation and real-time experimentation

The scientific method traditionally relies on two main approaches: hypothesis-driven and data-driven research. In hypothesis-driven research, scientists make specific predictions based on prior knowledge and design targeted experiments to test these hypotheses. For example, in microbiome studies, researchers might predict the presence of specific bacteria and design PCR primers for detection. In contrast, data-driven approaches collect comprehensive datasets without predetermined hypotheses, such as using metagenomics sequencing to identify all microbes present in a sample [91,92]. Both approaches complement each other, with data-driven methods often generating hypotheses that can be tested through targeted experiments [93,94].

Sophisticated frameworks for literature and data analysis are transforming hypothesis generation. FieldSHIFT [27], an in-context learning framework, uses large language models to generate candidate scientific research directions from existing published studies. These AI tools can identify patterns and relationships across different scientific domains, leading to novel research directions. In oncology, AI-driven algorithms analyzing extensive omics datasets have uncovered intricate details of cancer etiology, revealing insights potentially overlooked by traditional methods [95].

Beyond hypothesis generation, AI systems are now being applied to experimental methodologies, including live-cell imaging. Advances in AI-powered image analysis allow real-time tracking of cellular events, linking theoretical predictions with experimental validation. These methods can detect and quantify cellular dynamics, monitor cell morphology, and analyze complex 3D cultures with high accuracy and speed [54,96]. Deep learning algorithms further address challenges such as image drift and phototoxicity, enhancing spatial and temporal resolution [70]. These systems can track cells in diverse biological contexts, such as moving worm brains and beating zebrafish hearts, with notable precision [97].

In omics research, AI methods have advanced the analysis of complex biological datasets. Deep learning models can effectively handle high-dimensional omics data, integrating multiple data types to identify biomarkers and regulatory mechanisms [98]. Recent studies have employed AI to analyze liver disease by incorporating clinical information with molecular profiles for better diagnosis and treatment strategies [98]. As these models become more integrated into clinical research, the need for transparent and interpretable approaches has grown rapidly. Explainable AI (XAI) techniques have become crucial in omics analysis, with over 400 publications between 2010 and 2023 dedicated to making AI-derived insights more understandable and biologically meaningful [99]. This growing emphasis on interpretability sets the stage for broader applications of XAI in other areas of biology.

Model interpretability remains a central challenge in biological AI applications, as many systems still operate as ‘black boxes’ with limited transparency in their decision-making. This hampers biological validation, bias detection, and clinical translation. To improve explainability, techniques such as SHAP [100] and LIME have been used to prioritize biomarkers and interpret complex gene expression or cytokine-based predictions [101], while Grad-CAM++ and Layer-wise Relevance Propagation (LRP) enhance interpretability in histopathology [102] and multi-cancer prognostic models [103], respectively. Recent omics frameworks embed XAI techniques for automated feature attribution and pathway-level interpretation [104,105], while attention-based and prototype-driven models further promote interpretability by design [106,107].

Promoting deep integration between computational and experimental expertise is equally critical: establishing joint funding initiatives, interdisciplinary workshops, and integrated training programs can foster a common language and mutual understanding between data scientists and biologists. Placing computational researchers within biology labs – and biologists within computational teams – can help ensure that AI-generated hypotheses are not only statistically sound but also biologically plausible and experimentally testable.

## Challenges and future perspectives

High-quality datasets and standardized annotations remain critical challenges in computational cell biology [108]. Current research indicates that data quality has a significant impact on model performance, with issues such as batch effects and technical variations potentially leading to unreliable results [109], particularly in single-cell analysis where data heterogeneity can mask biological signals [110]. To address these issues, robust batch correction algorithms, such as scVI, have been developed and shown to improve cross-study integration and cell-type identification accuracy [111]. Additionally, community-driven efforts like the Human Cell Atlas are working toward developing standardized ontologies and annotation frameworks to improve consistency and data interoperability across laboratories [112]

Clinical translation of AI-driven discoveries remains a major hurdle. Regulatory barriers require model transparency and rigorous validation in diverse patient populations, often delaying adoption [46,94]. Beyond technical challenges, ethical and societal issues also deserve attention. Data privacy is critical when using patient-derived datasets, requiring compliance with regulations such as GDPR and HIPAA to maintain trust [113]. Moreover, AI models can perpetuate biases from training data, potentially reinforcing health disparities [114]. Additionally, the potential misuse of AI-generated biological insights underscores the need for strong governance and oversight [115,116].

The democratization of computational tools requires improved accessibility and training resources for researchers across different expertise levels. Platforms like the CZI AI Cell Models aim to make biologically relevant models and datasets easier to find, evaluate, and use [102]. Similarly, open-source tools such as DeepCell [31] and CellProfiler [29] offer accessible, cloud-based workflows and modular pipelines for image analysis. Commercial solutions like ZEISS arivis Cloud [117] further address computational resource challenges associated with analyzing large microscopy datasets.

Despite these advances, computational demands and scalability issues persist, particularly with large or multimodal datasets [118]. Current AI systems remain constrained by compute (high VRAM requirements, long training and inference times, and serving costs), scalability (batch effects, domain shift, and memory/IO bottlenecks across multi-institutional and multimodal data), and biological understanding (predictive features that lack mechanistic grounding, reliance on confounders, and limited ground truth for cell–cell interactions and perturbation responses). Overcoming these barriers will require more efficient architectures, streaming or distributed workflows, and closer integration with experimental validation to bridge prediction and biological insight. Addressing these challenges is also essential to ensure equitable global adoption of AI in biomedical research, as emphasized by recent Latin American initiatives that are building inclusive, AI-ready single-cell and spatial genomics datasets to counter regional and ancestral biases in model training [119,120].

Future directions in cell biology research point toward the development of comprehensive virtual cell models that integrate multiple data types and scales. Advances in spatial and single-cell multiomics and live-cell imaging enable reconstruction of dynamic cellular processes and tissue organization [42,69,121]. Recent developments in generative modeling, including diffusion models and flow matching, have provided state-of-the-art tools for simulating biological processes and temporal dynamics, enabling accurate prediction of cell or molecular state transitions over time [122]. Together, these technologies create a foundation for rapid hypothesis testing and for predicting cellular responses to diverse stimuli [122]. This integrative approach can lead to breakthroughs in biomedical research, personalized medicine, and drug discovery, particularly through the development of more sophisticated cell-scale models that can simulate complex biological processes.

## Conclusion

Computational approaches have fundamentally advanced cell biology research by enabling sophisticated analysis of complex biological data. Deep learning has advanced our understanding of cellular processes through precise image analysis, protein modeling, and multi-omics integration, offering comprehensive views of cellular function. These technological advances have contributed to progress in fundamental cell biology, drug development, and disease modeling.

The synergy between computational methods and experimental biology represents a new paradigm in cell research. This approach facilitates hypothesis generation and testing at unprecedented scales, with machine learning models uncovering patterns in complex datasets that guide experimental design and interpretation. The democratization of these tools, coupled with improvements in interpretability and validation methods, promises to make advanced computational approaches accessible to a broader scientific community. As these technologies mature, their application in cell biology promises advances in personalized medicine, drug development, and our understanding of cellular systems, while preserving the essential partnership between computational prediction and experimental validation.

**Perspectives**

- The incorporation of Artificial Intelligence is transforming cell biology by facilitating sophisticated analyses of complex biological data across areas such as cellular imaging and drug development. This technological progress marks a pivotal change in how cellular systems are studied.
- The current AI landscape in cell biology is marked by significant advances in single-cell resolution image analysis, protein structure prediction, multi-omics data integration, and cell behavior modeling, although challenges such as model interpretability and data quality requirements persist.
- The field is moving towards the development of comprehensive virtual cell models that integrate multiple data types and biological scales, enabling rapid hypothesis testing and prediction of cellular responses, with potential breakthroughs in personalized medicine and drug discovery through more sophisticated cell-scale simulations.

## Open Access

This article has been published open access under our Subscribe to Open programme, made possible through the support of our subscribing institutions, learn more here: https://portlandpress.com/pages/open_access_options_and_prices#conditional

## Abbreviations

ACC: accuracy
AI: artificial intelligence
AUC: area under the curve
AUROC: area under the receiver operating characteristic curve
CZI: Chan Zuckerberg Initiative
2D: two-dimensional
3D: three-dimensional
DEG: differentially expressed gene
DNA: deoxyribonucleic acid
GAN: generative adversarial network
GDPR: General Data Protection Regulation
GPU: graphics processing unit
HIPAA: Health Insurance Portability and Accountability Act
IO: input/output
IoU: intersection over union
LIME: Local Interpretable Model-agnostic Explanations
LRP: Layer-wise Relevance Propagation
ML: machine learning
PCR: polymerase chain reaction
PSC: pluripotent stem cell
RNA: ribonucleic acid
SHAP: Shapley Additive Explanations
SLA: service-level agreement
TEAD: TEA domain family member
VRAM: video random access memory
XAI: explainable artificial intelligence
scRNA-seq: single-cell RNA sequencing
